# Supplementary material for: Validating International Classification of Disease 10th Revision algorithms for identifying influenza and respiratory syncytial virus hospitalizations
Source: PLoS One. 2021 Jan 7;16(1):e0244746. doi: 10.1371/journal.pone.0244746 (PMC7790248; doi:10.1371/journal.pone.0244746)
Supplement: S1 Appendix — (DOCX) [file pone.0244746.s001.docx]

**S1 Appendix. Supplementary information for study methodology.**

**Table A. Ontario Health Insurance Plan (OHIP) eligibility criteria.**

| **To qualify for OHIP, individuals must meet all of the minimum qualifications listed below plus at least one of the additional requirements** | |
| --- | --- |
| 1. **Minimum Qualifications** | - Be physically in Ontario for 153 days in any 12-month period - Be physically in Ontario for at least 153 days of the first 183 days immediately after you began living in the province - Make Ontario their primary home |
| 1. **Additional Requirements** | Individuals:   - Are a Canadian citizen - Are an Indigenous person (registered under the federal *Indian* Act) - Are a permanent resident (formerly called a “landed immigrant”) - Have applied for permanent residence, and Immigration, Refugees and Citizenship Canada has confirmed that:   - You meet the eligibility requirements to apply   - You have not yet been denied - Are in Ontario on a valid work permit and are working full-time in Ontario for an Ontario employer, for at least six months   - Your spouse and any dependents also qualify if you do - Are in Ontario on a valid work permit under the federal Live-in Caregiver program - Are a convention refugee or other protected person (as defined by Immigration and Refugee Board of Canada) - Have a Temporary Resident Permit (only certain case types, eg. 86 through 95) - Are a clergy member who can legally stay in Canada and is ministering full time in Ontario for at least six months - Your spouse and any dependents also qualify if you do |

**Table B. Descriptions of ICD-10 codes included in influenza and RSV algorithm development.**

| **ICD-10 Code** | **Description** |
| --- | --- |
| **B34** | Viral infection, unspecified site |
| **B97** | Viral agents as the cause of disease classified to other chapters |
| **B97.4** | Respiratory Syncytial virus as the cause of diseases classified to other chapters |
| **J06** | Acute upper respiratory infections of multiple or unspecified sites |
| **J06.0** | Acute laryngopharyngitis |
| **J06.8** | Other acute respiratory infections of multiple sites |
| **J06.9** | Acute upper respiratory infection, unspecified |
| **J09** | Influenza due to identified novel influenza A virus |
| **J10** | Influenza, virus identified |
| **J10.0** | Influenza due to identified influenza virus |
| **J10.1** | Influenza with other respiratory manifestations, other influenza virus identified |
| **J10.8** | Influenza with other manifestations, other influenza virus identified |
| **J11** | Influenza, virus not identified |
| **J11.0** | Influenza with pneumonia, virus not identified |
| **J11.1** | Influenza with other respiratory manifestations, virus not identified |
| **J11.8** | Influenza with other manifestations, virus not identified |
| **J12** | Viral pneumonia, not elsewhere classified |
| **J12.1** | Respiratory syncytial virus pneumonia |
| **J12.8** | Other viral pneumonia |
| **J12.9** | Viral pneumonia, unspecified |
| **J18** | Bronchopneumonia, organism unspecified |
| **J18.0** | Bronchopneumonia, unspecified |
| **J18.8** | Other pneumonia, organism unspecified |
| **J18.9** | Pneumonia, unspecified |
| **J20** | Acute bronchitis |
| **J20.5** | Acute bronchitis due to respiratory syncytial virus |
| **J20.8** | Acute bronchitis due to other specified organisms |
| **J20.9** | Acute bronchitis, unspecified |
| **J21** | Acute bronchiolitis |
| **J21.0** | Acute bronchiolitis due to respiratory syncytial virus |
| **J21.8** | Acute bronchiolitis due to other specified organisms |
| **J21.9** | Acute bronchiolitis, unspecified |
| **J22** | Unspecified acute lower respiratory tract infection |

ICD-10, International Classification of Disease 10^th^ Revision; RSV, respiratory syncytial virus.
