# Supplementary material for: Validating International Classification of Disease 10th Revision algorithms for identifying influenza and respiratory syncytial virus hospitalizations
Source: PLoS One. 2021 Jan 7;16(1):e0244746. doi: 10.1371/journal.pone.0244746 (PMC7790248; doi:10.1371/journal.pone.0244746)
Supplement: S2 Appendix — (DOCX) [file pone.0244746.s002.docx]

**S2 Appendix. Supplementary results.**

**Table A. Validation of ICD-10 algorithms using most responsible diagnosis code only to identify hospitalized individuals with influenza infection.**

| **ICD-10 Algorithm** | **TP** | **FP** | **FN** | **TN** | **Sensitivity**  **(95% CI)** | **Specificity**  **(95% CI)** | **PPV (95% CI)** | **NPV (95% CI)** |
| --- | --- | --- | --- | --- | --- | --- | --- | --- |
| Influenza-specific codes^a^ | 6,675 | 319 | 8,079 | 68,565 | 0.45(0.44-0.46) | 1.00(0.99-1.00) | 0.95(0.95-0.96) | 0.89(0.89-0.90) |
| Influenza-specific + Influenza (virus not identified)^b^ | 7,585 | 567 | 7,169 | 68,317 | 0.51(0.51-0.52) | 0.99(0.99-0.99) | 0.93(0.92-0.94) | 0.91(0.90-0.91) |
| Influenza-specific + ARI of multiple/unspecified sites^c^ | 6,796 | 1,496 | 7,958 | 67,388 | 0.46(0.45-0.47) | 0.98(0.98-0.98) | 0.82(0.81-0.83) | 0.89(0.89-0.90) |
| Influenza-specific + viral pneumonia^d^ | 6,724 | 675 | 8,030 | 68,209 | 0.46(0.45-0.46) | 0.99(0.99-0.99) | 0.91(0.90-0.92) | 0.89(0.89-0.90) |
| Influenza-specific + bronchopneumonia^e^ | 7,177 | 8,759 | 7,577 | 60,125 | 0.49(0.48-0.49) | 0.87(0.87-0.88) | 0.45(0.44-0.46) | 0.89(0.89-0.89) |
| Influenza-specific + acute bronchitis^f^ | 6,701 | 578 | 8,053 | 68,306 | 0.45(0.45-0.46) | 0.99(0.99-0.99) | 0.92(0.91-0.93) | 0.89(0.89-0.90) |
| Influenza-specific + acute bronchiolitis^g^ | 6,709 | 1,533 | 8,045 | 67,351 | 0.45(0.45-0.46) | 0.98(0.98-0.98) | 0.81(0.81-0.82) | 0.89(0.89-0.90) |
| Influenza-specific + ARI of multiple sites + acute bronchitis + acute bronchiolitis | 7,645 | 2,040 | 7,109 | 66,844 | 0.52(0.51-0.53) | 0.97(0.97-0.97) | 0.79(0.78-0.80) | 0.90(0.90-0.91) |
| Influenza-specific + viral infection (unspecified site)^h^ | 6,750 | 1,236 | 8,004 | 67,648 | 0.46(0.45-0.47) | 0.98(0.98-0.98) | 0.85(0.84-0.85) | 0.89(0.89-0.90) |
| Influenza-specific + unspecified acute lower respiratory tract infection^i^ | 6,699 | 522 | 8,055 | 68,362 | 0.45(0.45-0.46) | 0.99(0.99-0.99) | 0.93(0.92-0.93) | 0.89(0.89-0.90) |
| Influenza-specific + all general ARI codes^j^ | 8,416 | 13,133 | 6,338 | 55,751 | 0.57(0.56-0.58) | 0.81(0.81-0.81) | 0.39(0.38-0.40) | 0.90(0.90-0.90) |

ICD-10, International Classification of Disease 10^th^ Revision; ARI, acute respiratory infection; TP, true positive; FP, false positive; FN, false negative; TN, true negative; PPV, positive predictive value; NPV, negative predictive value

a - Influenza-specific (virus identified) ICD-10 codes: J09, J10.0, J10.1, J10.8

b - Influenza (virus not identified) ICD-10 codes: J11.0, J11.1, J11.8

c - Acute upper respiratory infections of multiple unspecified sites (virus unspecified/not identified) ICD-10 codes: J06.0, J06.8, J06.9

d - Viral pneumonia (virus unspecified/not identified) ICD-10 codes: J12.8, J12.9

e - Bronchopneumonia (organism unspecified) ICD-10 codes: J18.0, J18.8, J18.9

f - Acute bronchitis (organism unspecified) ICD-10 codes: J20.8, J20.9

g - Acute bronchiolitis (organism unspecified) ICD-10 codes: J21.8, J21.9

h – Viral infection (unspecified site) ICD-10 code: B34

i - Unspecified acute lower respiratory tract infection ICD-10 code: J22

j - General ARI ICD-10 codes: J11.0 J11.1, J11.8, J06.0, J06.8, J06.9, J12.8, J12.9, J18.0, J18.8, J18.9, J20.8, J20.9, J21.8, J21.9, B34, J22

**Table B. Validation of ICD-10 algorithms using most responsible diagnosis code only to identify hospitalized individuals with RSV infection.**

| **ICD-10 Algorithm** | **TP** | **FP** | **FN** | **TN** | **Sensitivity**  **(95% CI)** | **Specificity**  **(95% CI)** | **PPV (95% CI)** | **NPV (95% CI)** |
| --- | --- | --- | --- | --- | --- | --- | --- | --- |
| RSV-specific codes^a^ | 2,393 | 253 | 3,221 | 55,250 | 0.43(0.41-0.44) | 1.00(0.99-1.00) | 0.90(0.89-0.92) | 0.94(0.94-0.95) |
| RSV-specific + ARI of multiple/unspecified sites^b^ | 2,623 | 960 | 2,991 | 54,543 | 0.47(0.45-0.48) | 0.98(0.98-0.98) | 0.73(0.72-0.75) | 0.95(0.95-0.95) |
| RSV-specific + Influenza (virus not identified)^c^ | 2,406 | 917 | 3,208 | 54,586 | 0.43(0.42-0.44) | 0.98(0.98-0.98) | 0.72(0.71-0.74) | 0.94(0.94-0.95) |
| RSV-specific + viral pneumonia^d^ | 2,431 | 489 | 3,183 | 55,014 | 0.43(0.42-0.45) | 0.99(0.99-0.99) | 0.83(0.82-0.85) | 0.95(0.94-0.95) |
| RSV-specific + bronchopneumonia^e^ | 2,672 | 6,128 | 2,942 | 49,375 | 0.48(0.46-0.49) | 0.89(0.89-0.89) | 0.30(0.29-0.31) | 0.94(0.94-0.95) |
| RSV-specific + acute bronchitis^f^ | 2,402 | 408 | 3,212 | 55,095 | 0.43(0.41-0.44) | 0.99(0.99-0.99) | 0.85(0.84-0.87) | 0.94(0.94-0.95) |
| RSV-specific + acute bronchiolitis^g^ | 2,600 | 730 | 3,014 | 54,773 | 0.46(0.45-0.48) | 0.99(0.99-0.99) | 0.78(0.77-0.79) | 0.95(0.95-0.95) |
| RSV-specific + ARI of multiple sites + acute bronchitis + acute bronchiolitis | 2,839 | 1,592 | 2,775 | 53,911 | 0.51(0.49-0.52) | 0.97(0.97-0.97) | 0.64(0.63-0.65) | 0.95(0.95-0.95) |
| RSV-specific + viral infection (unspecified site)^h^ | 2,450 | 871 | 3,164 | 54,632 | 0.44(0.42-0.45) | 0.98(0.98-0.99) | 0.74(0.72-0.75) | 0.95(0.94-0.95) |
| RSV-specific + unspecified acute lower respiratory tract infection^i^ | 2,428 | 355 | 3,186 | 55,148 | 0.43(0.42-0.45) | 0.99(0.99-0.99) | 0.87(0.86-0.88) | 0.95(0.94-0.95) |
| RSV-specific + all general ARI codes^j^ | 3,261 | 9,087 | 2,353 | 46,416 | 0.58(0.57-0.59) | 0.84(0.83-0.84) | 0.26(0.26-0.27) | 0.95(0.95-0.95) |

ICD-10, International Classification of Disease 10^th^ Revision; RSV, respiratory syncytial virus; ARI, acute respiratory infection; TP, true positive; FP, false positive; FN, false negative; TN, true negative; PPV, positive predictive value; NPV, negative predictive value.

a - RSV-specific (virus identified) ICD-10 codes: J12.1, J20.5, J21.0, B97.4

b - Acute upper respiratory infections of multiple unspecified sites (virus unspecified/not identified) ICD-10 codes: J06.0, J06.8, J06.9

c - Influenza (virus not identified) ICD-10 codes: J11.0, J11.1, J11.8

d - Viral pneumonia (virus unspecified/not identified) ICD-10 codes: J12.8, J12.9

e - Bronchopneumonia (organism unspecified) ICD-10 codes: J18.0, J18.8, J18.9

f - Acute bronchitis (organism unspecified) ICD-10 codes: J20.8, J20.9

g - Acute bronchiolitis (organism unspecified) ICD-10 codes: J21.8, J21.9

h – Viral infection (unspecified site) ICD-10 code: B34

i - Unspecified acute lower respiratory tract infection ICD-10 code: J22

j - General ARI ICD-10 codes: J11.0 J11.1, J11.8, J06.0, J06.8, J06.9, J12.8, J12.9, J18.0, J18.8, J18.9, J20.8, J20.9, J21.8, J21.9, B34, J22

**Table C. Comparison of Youden’s Index and Cohen’s Kappa for influenza algorithms applied to most responsible diagnosis versus all diagnosis codes on a patient’s hospital record.**

| **ICD-10 Algorithm** | **Most Responsible Diagnosis Code** | | **All Diagnosis Codes** | |
| --- | --- | --- | --- | --- |
|  | **Youden’s Index** | **Cohen’s Kappa** | **Youden’s Index** | **Cohen’s Kappa** |
| Influenza-specific codes^a^ | 0.448 | 0.564 | 0.719 | 0.790 |
| Influenza-specific + Influenza (virus not identified)^b^ | 0.506 | 0.614 | 0.813 | 0.842 |
| Influenza-specific + ARI of multiple/unspecified sites^c^ | 0.439 | 0.530 | 0.695 | 0.703 |
| Influenza-specific + viral pneumonia^d^ | 0.446 | 0.555 | 0.714 | 0.767 |
| Influenza-specific + bronchopneumonia^e^ | 0.359 | 0.348 | 0.509 | 0.361 |
| Influenza-specific + acute bronchitis^f^ | 0.446 | 0.557 | 0.715 | 0.772 |
| Influenza-specific + acute bronchiolitis^g^ | 0.432 | 0.523 | 0.701 | 0.737 |
| Influenza-specific + ARI of multiple sites + acute bronchitis + acute bronchiolitis | 0.489 | 0.565 | 0.789 | 0.773 |
| Influenza-specific + viral infection (unspecified site)^h^ | 0.440 | 0.536 | 0.702 | 0.728 |
| Influenza-specific + unspecified acute lower respiratory tract infection^i^ | 0.446 | 0.559 | 0.716 | 0.777 |
| Influenza-specific + all general ARI codes^j^ | 0.380 | 0.322 | 0.529 | 0.319 |

ICD-10, International Classification of Disease 10^th^ Revision; RSV, respiratory syncytial virus; ARI, acute respiratory infection.

a - Influenza-specific (virus identified) ICD-10 codes: J09, J10.0, J10.1, J10.8

b - Influenza (virus not identified) ICD-10 codes: J11.0, J11.1, J11.8

c - Acute upper respiratory infections of multiple unspecified sites (virus unspecified/not identified) ICD-10 codes: J06.0, J06.8, J06.9

d - Viral pneumonia (virus unspecified/not identified) ICD-10 codes: J12.8, J12.9

e - Bronchopneumonia (organism unspecified) ICD-10 codes: J18.0, J18.8, J18.9

f - Acute bronchitis (organism unspecified) ICD-10 codes: J20.8, J20.9

g - Acute bronchiolitis (organism unspecified) ICD-10 codes: J21.8, J21.9

h – Viral infection (unspecified site) ICD-10 code: B34

i - Unspecified acute lower respiratory tract infection ICD-10 code: J22

j - General ARI ICD-10 codes: J11.0 J11.1, J11.8, J06.0, J06.8, J06.9, J12.8, J12.9, J18.0, J18.8, J18.9, J20.8, J20.9, J21.8, J21.9, B34, J22

**Table D. Comparison of Youden’s Index and Cohen’s Kappa for RSV algorithms applied to most responsible diagnosis versus all diagnosis codes on a patient’s hospital record.**

| **ICD-10 Algorithm** | **Most Responsible Diagnosis Code** | | **All Diagnosis Codes** | |
| --- | --- | --- | --- | --- |
|  | **Youden’s Index** | **Cohen’s Kappa** | **Youden’s Index** | **Cohen’s Kappa** |
| RSV-specific codes^a^ | 0.422 | 0.553 | 0.684 | 0.766 |
| RSV-specific + ARI of multiple/unspecified sites^b^ | 0.450 | 0.537 | 0.682 | 0.655 |
| RSV-specific + Influenza (virus not identified)^c^ | 0.412 | 0.505 | 0.666 | 0.675 |
| RSV-specific + viral pneumonia^d^ | 0.424 | 0.541 | 0.686 | 0.733 |
| RSV-specific + bronchopneumonia^e^ | 0.366 | 0.291 | 0.516 | 0.260 |
| RSV-specific + acute bronchitis^f^ | 0.421 | 0.542 | 0.680 | 0.736 |
| RSV-specific + acute bronchiolitis^g^ | 0.450 | 0.551 | 0.715 | 0.746 |
| RSV-specific + ARI of multiple sites + acute bronchitis + acute bronchiolitis | 0.477 | 0.527 | 0.707 | 0.624 |
| RSV-specific + viral infection (unspecified site)^h^ | 0.421 | 0.515 | 0.673 | 0.674 |
| RSV-specific + unspecified acute lower respiratory tract infection^i^ | 0.426 | 0.551 | 0.683 | 0.750 |
| RSV-specific + all general ARI codes^j^ | 0.417 | 0.271 | 0.510 | 0.209 |

ICD-10, International Classification of Disease 10^th^ Revision; RSV, respiratory syncytial virus; ARI, acute respiratory infection.

a - RSV-specific (virus identified) ICD-10 codes: J12.1, J20.5, J21.0, B97.4

b - Acute upper respiratory infections of multiple unspecified sites (virus unspecified/not identified) ICD-10 codes: J06.0, J06.8, J06.9

c - Influenza (virus not identified) ICD-10 codes: J11.0, J11.1, J11.8

d - Viral pneumonia (virus unspecified/not identified) ICD-10 codes: J12.8, J12.9

e - Bronchopneumonia (organism unspecified) ICD-10 codes: J18.0, J18.8, J18.9

f - Acute bronchitis (organism unspecified) ICD-10 codes: J20.8, J20.9

g - Acute bronchiolitis (organism unspecified) ICD-10 codes: J21.8, J21.9

h – Viral infection (unspecified site) ICD-10 code: B34

i - Unspecified acute lower respiratory tract infection ICD-10 code: J22

j - General ARI ICD-10 codes: J11.0 J11.1, J11.8, J06.0, J06.8, J06.9, J12.8, J12.9, J18.0, J18.8, J18.9, J20.8, J20.9, J21.8, J21.9, B34, J22

**Table E. Validation of top-performing ICD-10 algorithms by month of hospital admission.**

| **ICD-10 Algorithm** | **TP** | **FP** | **FN** | **TN** | **Sensitivity**  **(95% CI)** | **Specificity**  **(95% CI)** | **PPV (95% CI)** | **NPV (95% CI)** |
| --- | --- | --- | --- | --- | --- | --- | --- | --- |
| **FLU1 Algorithm^a^** |  |  |  |  |  |  |  |  |
| Nov | 126 | 19 | 70 | 7,023 | 0.64(0.57-0.71) | 1.00(1.00-1.00) | 0.87(0.80-0.92) | 0.99(0.99-0.99) |
| Dec | 1,485 | 91 | 620 | 10,643 | 0.71(0.69-0.72) | 0.99(0.99-0.99) | 0.94(0.93-0.95) | 0.95(0.94-0.95) |
| Jan | 3,363 | 181 | 1,163 | 14,821 | 0.74(0.73-0.76) | 0.99(0.99-0.99) | 0.95(0.94-0.96) | 0.93(0.92-0.93) |
| Feb | 2,759 | 158 | 1,004 | 11,503 | 0.73(0.72-0.75) | 0.99(0.98-0.99) | 0.95(0.94-0.95) | 0.92(0.91-0.92) |
| Mar | 2,012 | 127 | 711 | 10,972 | 0.74(0.72-0.76) | 0.99(0.99-0.99) | 0.94(0.93-0.95) | 0.94(0.93-0.94) |
| Apr | 834 | 48 | 308 | 7,884 | 0.73(0.70-0.76) | 0.99(0.99-1.00) | 0.95(0.93-0.96) | 0.96(0.96-0.97) |
| May | 176 | 29 | 123 | 5,385 | 0.59(0.53-0.65) | 0.99(0.99-1.00) | 0.86(0.80-0.90) | 0.98(0.97-0.98) |
| **FLU2 Algorithm^b^** |  |  |  |  |  |  |  |  |
| Nov | 147 | 49 | 49 | 6,993 | 0.75(0.68-0.81) | 0.99(0.99-0.99) | 0.75(0.68-0.81) | 0.99(0.99-0.99) |
| Dec | 1,769 | 181 | 336 | 10,553 | 0.84(0.82-0.86) | 0.98(0.98-0.99) | 0.91(0.89-0.92) | 0.97(0.97-0.97) |
| Jan | 3,845 | 365 | 681 | 14,637 | 0.85(0.84-0.86) | 0.98(0.97-0.98) | 0.91(0.90-0.92) | 0.96(0.95-0.96) |
| Feb | 3,099 | 269 | 664 | 11,392 | 0.82(0.81-0.84) | 0.98(0.97-0.98) | 0.92(0.91-0.93) | 0.94(0.94-0.95) |
| Mar | 2,232 | 203 | 491 | 10,896 | 0.82(0.80-0.83) | 0.98(0.98-0.98) | 0.92(0.90-0.93) | 0.96(0.95-0.96) |
| Apr | 947 | 88 | 195 | 7,844 | 0.83(0.81-0.85) | 0.99(0.99-0.99) | 0.92(0.90-0.93) | 0.98(0.97-0.98) |
| May | 206 | 46 | 93 | 5,368 | 0.69(0.63-0.74) | 0.99(0.99-0.99) | 0.82(0.76-0.86) | 0.98(0.98-0.99) |
| **RSV1 Algorithm^c^** |  |  |  |  |  |  |  |  |
| Nov | 308 | 26 | 111 | 5,240 | 0.74(0.69-0.78) | 1.00(0.99-1.00) | 0.92(0.89-0.95) | 0.98(0.98-0.98) |
| Dec | 974 | 79 | 320 | 8,681 | 0.75(0.73-0.78) | 0.99(0.99-0.99) | 0.93(0.91-0.94) | 0.96(0.96-0.97) |
| Jan | 1,130 | 103 | 545 | 13,459 | 0.67(0.65-0.70) | 0.99(0.99-0.99) | 0.92(0.90-0.93) | 0.96(0.96-0.96) |
| Feb | 757 | 108 | 341 | 10,777 | 0.69(0.66-0.72) | 0.99(0.99-0.99) | 0.88(0.85-0.90) | 0.97(0.97-0.97) |
| Mar | 510 | 62 | 260 | 10,056 | 0.66(0.63-0.70) | 0.99(0.99-1.00) | 0.89(0.86-0.92) | 0.97(0.97-0.98) |
| Apr | 202 | 25 | 156 | 6,887 | 0.56(0.51-0.62) | 1.00(0.99-1.00) | 0.89(0.84-0.93) | 0.98(0.97-0.98) |
| **RSV2 Algorithm^d^** |  |  |  |  |  |  |  |  |
| Nov | 309 | 45 | 110 | 5,221 | 0.74(0.69-0.78) | 0.99(0.99-0.99) | 0.87(0.83-0.91) | 0.98(0.98-0.98) |
| Dec | 977 | 98 | 317 | 8,662 | 0.76(0.73-0.78) | 0.99(0.99-0.99) | 0.91(0.89-0.93) | 0.96(0.96-0.97) |
| Jan | 1,133 | 165 | 542 | 13,397 | 0.68(0.65-0.70) | 0.99(0.99-0.99) | 0.87(0.85-0.89) | 0.96(0.96-0.96) |
| Feb | 762 | 144 | 336 | 10,741 | 0.69(0.67-0.72) | 0.99(0.98-0.99) | 0.84(0.82-0.86) | 0.97(0.97-0.97) |
| Mar | 512 | 98 | 258 | 10,020 | 0.66(0.63-0.70) | 0.99(0.99-0.99) | 0.84(0.81-0.87) | 0.97(0.97-0.98) |
| Apr | 203 | 48 | 155 | 6,864 | 0.57(0.51-0.62) | 0.99(0.99-0.99) | 0.81(0.75-0.86) | 0.98(0.97-0.98) |

ICD-10, International Classification of Disease 10^th^ Revision; RSV, respiratory syncytial virus; TP, true positive; FP, false positive; FN, false negative; TN, true negative; PPV, positive predictive value; NPV, negative predictive value.

a - Influenza-specific ICD-10 codes with virus identified: J09, J10.0, J10.1, J10.8

b - Influenza-specific ICD-10 codes with and without virus identified: J09, J10.0, J10.1, J10.8, J11.0, J11.1, J11.8

c - RSV-specific ICD-10 codes with virus identified: J12.1, J20.5, J21.0, B97.4

d - RSV-specific ICD-10 codes with virus identified + unspecified acute lower respiratory tract infection ICD-10 code: J12.1, J20.5, J21.0, B97.4, J22
